# The *GDF5* rs143384 Polymorphism Is Associated with the Severity of Knee Osteoarthritis and Shorter Stature in Female Brazilian Patients: A Cross-Sectional Study

**DOI:** 10.3390/genes16121520

**Published:** 2025-12-18

**Authors:** Jamila Alessandra Perini, Igor Stefano Menescal Pedrinha, Lucas Rafael Lopes, Phelippe Augusto Valente Maia, Jéssica Vilarinho Cardoso, Eduardo Branco de Sousa

**Affiliations:** 1 Research Laboratory of Pharmaceutical Sciences (LAPESF), Pharmacy Department, Rio de Janeiro of State University (UERJ), Rio de Janeiro 23070-200, Brazil; lopes.rlucas02@gmail.com (L.R.L.); jessica_vilarinho@yahoo.com.br (J.V.C.); 2 Knee Surgery Center, National Institute of Traumatology and Orthopaedics (INTO), Rio de Janeiro 20940-070, Brazil; igorsmpedrinha@yahoo.com.br (I.S.M.P.); phemaia@yahoo.com.br (P.A.V.M.); 3 General and Specialized Surgery Department, Faculty of Medicine, Fluminense Federal University (UFF), Rio de Janeiro 24033-900, Brazil; eduardobranco@id.uff.br

**Keywords:** cartilage degeneration, synovial inflammation, growth differentiation factor, transforming growth factor beta, genetic variation, single nucleotide polymorphism, risk factor

## Abstract

Background/Objectives: Knee osteoarthritis (KOA) is a multifactorial and degenerative disease. Growth differentiation factor 5 (*GDF5*) polymorphism rs143384 G > A is associated with reduced gene expression and musculoskeletal pathologies. This study aimed to evaluate the association between this functional polymorphism and clinical variability and disease severity among patients with KOA in an admixed population. Methods: This cross-sectional observational study enrolled 224 Brazilian patients with KOA, who were evaluated and classified according to disease severity. Results: The median age was 64 (44–84) years; 75.9% of the patients were female, 50.9% were shorter than 1.60 m, and 67.4% were obese or morbidly obese. The disease severity distribution was 64.7% grades I–III and 35.3% IV–V. Patients with KOA who were over 70 years had significantly more advanced grades (OR = 9.3; 95% CI = 3.4–26), in either female group (OR = 8.2; 95% CI = 2.6–26). The minor allele frequency of the *GDF5* rs143384 A variant was 41.7% in the overall KOA case group, increasing with disease severity (39.7% in grades I–III versus 45.6% in IV–V). After adjusting for the confounding factors (age and BMI) the *GDF5* GA + AA genotype was significantly associated with higher KOA severity IV–V in female patients (OR = 2.5; 95% CI = 1.2–5.3). Additionally, the mean height of female KOA patients with the *GDF5* GA + AA genotype (1.56 ± 0.07 m) was significantly shorter than that of patients with the GG genotype (1.59 ± 0.08 m). Conclusions: The *GDF5* rs143384 polymorphism was associated with greater KOA severity and shorter stature in female patients. These results suggest that this variant may contribute to phenotypic variability in patients with knee osteoarthritis, helping to refine clinical characterization and stratification in this population, contributing to personalized diagnoses and guiding future changes in treatment guidelines for knee osteoarthritis.

## 1. Introduction

Osteoarthritis is a chronic, progressive, and painful inflammatory disease characterized by cartilage degeneration, structural alterations in joint tissues, and synovial inflammation. Knee osteoarthritis (KOA) is the most common form of the disease due to the knee’s weight-bearing function and constant exposure to mechanical stress [1]. In 2021, the global prevalence of KOA was 4748.73, with an incidence rate of 390.88 per 100,000 people. These rates were higher in women (5854.31 and 465.41, respectively) than in men [2]. Women, particularly those who have reached menopause, are more susceptible to KOA due to hormonal and biomechanical changes. Advancing age also contributes to joint degeneration and impaired tissue repair [3]. Additionally, greater adult height has been associated with an increased risk of KOA [4].

KOA diagnosis is primarily clinical and supported by imaging examinations. Its treatment is symptomatic and includes lifestyle modifications, pharmacotherapy and, in advanced cases, surgical interventions [5]. This disease poses a significant challenge to public health systems worldwide, as it negatively affects individuals’ quality of life and functional capacity and imposes a high economic and social burden [6].

The etiology of KOA is multifactorial and not yet fully understood. Genetic factors are believed to play a substantial role, with an estimated heritability of 39% [7], potentially increasing to 53% in more severe cases and up to 80% among women over 50 years old [8]. Genome-wide association studies (GWAS) have identified over 80 loci associated with KOA, among which the *GDF5* (growth differentiation factor 5) gene stands out due to its role in the development, maintenance, and repair of synovial joints [9,10,11]. *GDF5* is located on chromosome 20q11.2, contains two exons, and encodes a signaling protein of the transforming growth factor beta (TGF-β) superfamily. It is also a highly polymorphic gene (https://www.ncbi.nlm.nih.gov/gtr/genes/8200/, accessed on 5 December 2025). Among its single nucleotide polymorphisms (SNPs), *GDF5* rs143384 is of particular interest as a marker associated with KOA [9,12,13,14] and other musculoskeletal pathologies [15,16,17,18]. This SNP, located in the 5′ untranslated region (5′-UTR), is associated with the A allele and reduced *GDF5* expression, which may impair the maintenance and repair of articular structures [12,14]. Therefore, this study aimed to evaluate the association between the *GDF5* rs143384 and clinical variability and disease severity among Brazilian patients with KOA, considering the functional importance of this SNP and its role in the disease.

## 2. Materials and Methods

This cross-sectional observational study enrolled 224 patients with KOA who were treated by the Knee Surgery Service at the National Institute of Traumatology and Orthopaedics in Rio de Janeiro, Brazil, between 1 October 2021 and 31 December 2024. All patients provided written informed consent for research involving human subjects in accordance with the Declaration of Helsinki. The study was approved by the Institutional Research Ethics Committee (CAAE #51922121.2.0000.5273/2021).

The knee radiographs were evaluated and classified according to the modified Ahlbäck classification by Keyes and Goodfellow [19]. Sociodemographic and anthropometric data, including sex, age, weight, and height, as well as information on a positive history of rheumatoid arthritis, gout, lupus and other types of arthritis, and a diagnosis of osteoarthritis in other joints, was obtained through interviews and an active search of medical records. This information was recorded using a standardized data collection instrument. Patients of both sexes aged over 40 years with a diagnosis of primary KOA established through clinical and radiographic examination were included in the study [20]. Patients were excluded if there was no biological material available for polymorphism analysis, or if they had previously undergone surgery, experienced an infection, or had neoplasia in the affected knee.

Oral mucosal epithelium was collected using a swab for subsequent genomic DNA extraction using a Qiagen (Hilden, Germany) extraction kit according to the manufacturer’s recommendations. Genotyping analysis of the *GDF5* rs143384 G > A SNP was performed using a Real-Time Polymerase Chain Reaction (PCR) with a TaqMan™ system. The PCR was carried out in a final volume of 8 µL containing 1 µL of DNA, 3.5 µL of TaqMan Universal Master Mix (Applied Biosystems, Foster City, CA, USA), 0.2 µL of the TaqMan™ SNP Genotyping Assay for *GDF5* rs143384 G > A (Assay ID: C_5991441, Thermo Fisher Scientific, Carlsbad, CA, USA) and sterile H_2_O to make up the final volume. The reaction included two negative controls (without DNA) and two positive controls for each of the three genotypes (wild-type, heterozygote, and variant homozygote). DNA amplification was analyzed using a 7500 Real-Time System (Applied Biosystems). The thermal cycling conditions required for DNA amplification have been described elsewhere [18].

A priori power analysis was performed using G*Power 3.1 to estimate the required sample size for detecting differences in genotype frequencies between groups, assuming α = 0.05 and 80% power. The normality of the distribution was assessed using the Kolmogorov–Smirnov test. As the continuous variables (age, height, and BMI) were not normally distributed (*p* < 0.01), they were presented as medians and interquartile ranges (IQRs). Group comparisons for these variables were performed using the Mann–Whitney U test. Nominal data were expressed as proportions and analyzed using the chi-squared (χ^2^) test or Fisher’s exact test when appropriate. To determine whether the genotypes deviated from Hardy–Weinberg equilibrium (HWE), the observed and expected genotype frequencies were compared using a chi-squared (χ^2^) goodness-of-fit test. The allele and genotype distributions for the *GDF5* rs143384 SNP were determined by gene counting, and differences in frequencies between the two groups were assessed using either the chi-squared test or Fisher’s exact test.

For the association analysis, the dependent variable (i.e., the outcome) was disease severity, as defined by the Ahlbäck classification system. Patients were categorized into two groups: grades I–III and IV–V. The independent variable, age, was categorized into quartiles based on its distribution. Body mass index (BMI) was classified according to National Institutes of Health (NIH) criteria as follows: normal or overweight (BMI < 30 kg/m^2^); obesity (BMI 30–34.9 kg/m^2^); and morbid obesity (BMI ≥ 35 kg/m^2^) [21]. The association between the *GDF5* rs143384 SNP and KOA severity was estimated using odds ratios (ORs) and 95% confidence intervals (CIs) via binary logistic regression. The final model was constructed based on statistical significance from univariate analyses and biological relevance, using a stepwise variable selection method. Model fit was assessed using the Hosmer–Lemeshow test. Additionally, the association between the SNP and height was evaluated using the Mann–Whitney U test to determine its discriminatory ability between groups. All analyses were conducted using the Statistical Package for the Social Sciences, version 20.0 (SPSS, IBM Corp., Armonk, NY, USA), with statistical significance set at *p* < 0.05.

## 3. Results

The sample consisted of 224 patients with osteoarthritis. The median age was 64 years (range: 44–84 years; interquartile range (IQR): 11.8). Of these patients, 170 (75.9%) were female; 114 (50.9%) were shorter than 1.60 m; 151 (67.4%) were obese or morbidly obese; and 13 (5.8%) had a history of trauma prior to diagnosis. The distribution of disease severity according to the Ahlbäck classification was as follows: Grade I: 46 patients (20.5%); Grade II: 63 patients (28.1%); Grade III: 36 patients (16.1%); Grade IV: 46 patients (20.5%); Grade V: 33 patients (14.7%). Table 1 presents the epidemiological profile stratified by Ahlbäck grades (I–III vs. IV–V), showing that patients with more advanced grades were older. No other characteristics differed between groups. A similar pattern was observed when the analysis was restricted to female patients (n = 170) (Table 1).

**Figure 1:**
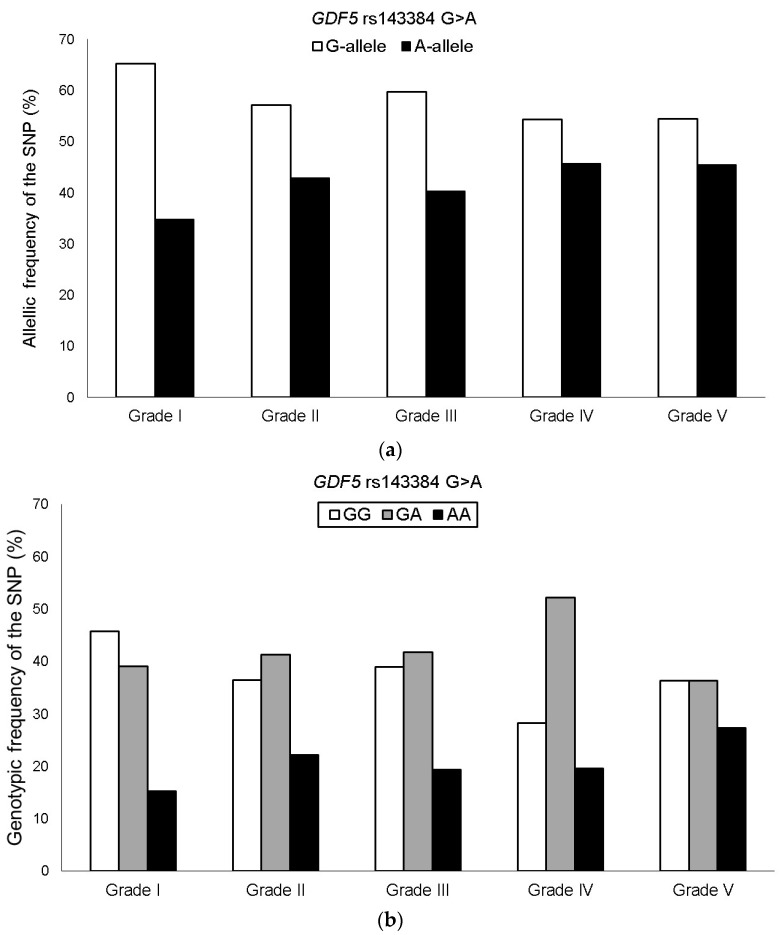
Allelic (**a**) and genotypic (**b**) frequencies of the *GDF5* rs143384 G > A SNP among KOA patient groups stratified by Ahlbäck classification grades.

The genotyping success rate for the *GDF5* rs143384 G > A SNP was 100%, and the genotypic distribution was in HWE among KOA patients. The minor allele frequency (MAF) of the rs143384 A in the Brazilian KOA group was 41.7%, with the following genotypes: GG = 37.1%, GA = 42.4%, and AA = 20.5%. The frequency of the A allele increased with higher Ahlbäck classification grades. However, no significant associations were observed when comparing severity groups in the overall KOA sample (Figure 1).

In the stratified analysis of female KOA patients, the frequency of the minor allele (*GDF5* rs143384 A) increased progressively with disease severity. On average, there was an increment of approximately 4–5% between grades, ranging from 34.4% in grade I to 52.2% in grade V. Additionally, after adjusting for the confounding factors of age and BMI, the heterozygous genotype and the codominant model (*GDF5* rs143384 GA + AA) were significantly associated with greater severity among KOA patients when comparing Ahlbäck classification grades I–III vs. IV–V, I–II vs. III–V, and I–II vs. IV–V (Table 2). No significant association of the *GDF5* rs143384 SNP was observed when grade III was analyzed separately compared to the other severity grades. In addition, the genotype × age interaction was tested by introducing a multiplicative term in the logistic regression model. The interaction term was not statistically significant (*p* = 0.49), indicating no evidence of effect modification by age.

**Figure 2:**
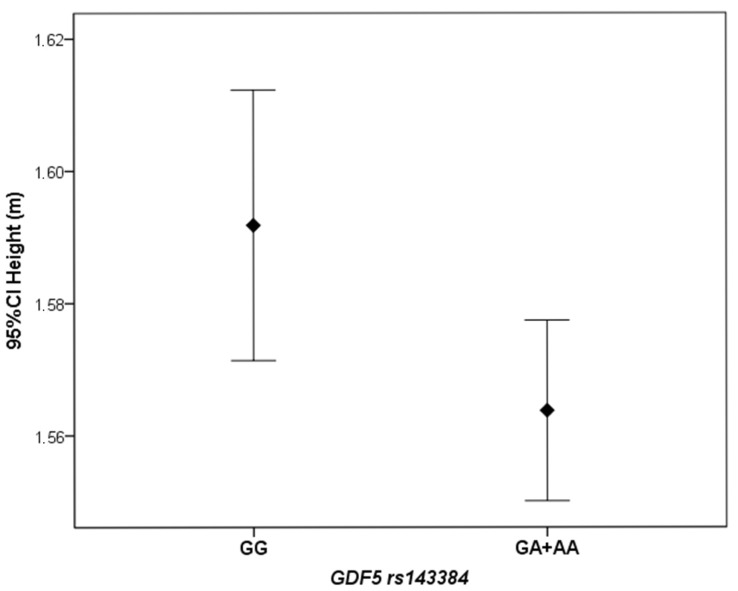
Difference in mean height between female KOA patients carrying the *GDF5* rs143384 GA or AA genotypes, compared to those with the GG genotype.

Female KOA patients carrying the *GDF5* rs143384 GA or AA genotypes (codominant model) had significantly lower height than those with the GG genotype (*p* = 0.04). The median height of individuals carrying the A allele was 1.56 m (IQR: 0.09), whereas the median height of individuals with the GG genotype was 1.59 m (IQR: 0.10). Similarly, the mean height was higher in the GG group (1.59 ± 0.08 m) than in the GA + AA group (1.56 ± 0.07 m), as shown in Figure 2.

## 4. Discussion

KOA is a complex degenerative disorder influenced by genetic and environmental factors. However, studies examining genetic contributions in highly admixed populations, such as in Brazil, remain scarce [1]. Among the genes implicated in KOA, *GDF5* is of particular importance due to its role in joint development, maintenance, and repair [9,10,11]. In this context, our study explores the association between the rs143384 SNP in *GDF5* and KOA severity and clinical variability in patients from a public orthopedic referral hospital. Our results suggest that female patients with the *GDF5* rs143384 GA or AA genotypes have more severe KOA and are slightly shorter than those with the GG genotype. Furthermore, the frequency of the A allele increased progressively with higher Ahlbäck grades, suggesting a potential role for this SNP in disease progression among Brazilian patients.

Of the 224 KOA patients enrolled in the study, nearly 76% were female and 67% obese, with a median age of 64 years. This reflects the general epidemiology of KOA, which predominantly affects women [22]. Obesity is the most prevalent preventable risk factor for osteoarthritis, with effects ranging from biomechanical overload to complex metabolic and inflammatory pathways. The risk of KOA, which is the joint most susceptible to obesity-related stress, is increased by between 1.3- to 6.0-fold, with a more pronounced effect in women [23], which corroborates our findings. Moreover, women with moderate to severe KOA had a higher prevalence of the *GDF5* rs143384 GA + AA genotype.

The *GDF5* rs143384 polymorphism is one of the most widely studied in relation to osteoarthritis [13,24,25]. The *GDF5* gene plays a key role in skeletal and joint development. Specific variants of this gene, particularly *GDF5* rs143384 and rs143383, have been linked to conditions such as osteoarthritis, congenital hip dislocation, and chronic pain in genome-wide association studies [25]. Specifically, the *GDF5* rs143384 allele variant A has been associated with knee pain [17,26,27], hip dysplasia [28,29] and hand osteoarthritis [13]. On the other hand, a systematic review found that the rs143383 was associated with osteoarthritis in both men and women. However, the analysis of hip osteoarthritis remained inconclusive due to the high statistical heterogeneity, indicating a need for further research in this area [30]. Furthermore, another study concluded that the *GDF5* rs143383 SNP is associated with KOA risk in Caucasians, but not in Asians [31]. It is worth noting that the two SNPs, rs143383 and rs143384, are in strong linkage disequilibrium (r^2^ = 0.82). This indicates that they are frequently co-inherited and likely represent the same association signal [14,32]. These findings emphasize the importance of investigating *GDF5* variants as potential genetic biomarkers of KOA-related phenotypic variability and severity, particularly in the highly admixed populations such as the Brazilian population [18].

In this study, female KOA patients carrying the *GDF5* rs143384 GA or AA genotypes (codominant model) were shorter than those with the *GDF5* rs143384 GG genotype. The height of humans and other characteristics of body size and shape have been extensively studied in large genome-wide association studies, yielding many associated loci [33,34]. The size and shape of bones are an important component of human height and body form. Height is directly influenced by bone size, and bone dimensions and shape also determine their strength and susceptibility to fractures [35], and the tendency to develop osteoarthritis. Vaes et al., (2009) reported that the C allele of the *GDF5* rs143383 was associated with a taller stature [36]. In contrast, Sanna et al., (2008) found that the *GDF5* A allele, which is associated with an increased risk of osteoarthritis, correlated with decreased height [37]. Similarly, Wu et al., (2012) demonstrated that the A alleles derived from *GDF5* rs143383 and *GDF5* rs143384 were associated with reduced height [32]. More recently, Styrkarsdottir et al., (2019) confirmed the association of *GDF5* rs143384 with both height and bone area [16]. This finding reinforces the role of *GDF5* in skeletal development and is in line with the results of the present study.

The association found in our study may be functionally explained by the molecular consequences of the A allele on *GDF5* expression. The A allele of the rs143384 SNP reduces *GDF5* expression by altering transcription factor binding and CpG methylation mechanisms [38,39]. Reduced levels of *GDF5* impair the proliferation and differentiation of chondrocytes, decrease synthesis of the extracellular matrix (type II collagen and aggrecan) and increase the activity of catabolic enzymes (MMPs and ADAMTS). This leads to diminished cartilage repair and greater mechanical vulnerability [40]. Consequently, this molecular dysregulation promotes progressive cartilage degeneration and may contribute to increased risk and severity of osteoarthritis, rather than establishing a direct causal mechanism. It also may influence endochondral ossification and longitudinal bone growth [32,41], which could help explain the shorter stature observed in carriers of the A allele, particularly among women with knee osteoarthritis. In summary, these data reinforce the potential of *GDF5* rs143384 as a molecular biomarker of cartilage-related biological pathways and KOA phenotypic variability. This provides additional context for understanding the variability in disease onset, progression and clinical presentation among individuals [18].

In our study, the codominant model (*GDF5* rs143384 GA + AA) was associated with greater KOA severity following adjustment for BMI and age. Novakov et al. [14] found that, in obese individuals (BMI ≥ 30), the G allele of *GDF5* rs143384 acted as a protective factor, significantly reducing the risk of KOA. However, in non-obese individuals (BMI < 30), the same *GDF5* rs143384 G allele acted as a risk factor, increasing the likelihood of developing the disease. This phenomenon may be attributed to the regulatory effect of the SNP on *GDF5* expression within adipose tissue, highlighting a critical gene–environment interaction whereby obesity alters genetic predisposition to KOA [14]. Similarly, Jia et al., suggested that the *GDF5* rs143383 polymorphism was associated with KOA susceptibility and had a protective effect against KOA in Caucasian, Asian, and African populations [42]. However, the authors did not refer to the influence of BMI, as they did in the other study. In addition, Witoonpanich et al., successfully demonstrated that *GDF5* is expressed in the synovial membrane, with higher expression levels observed in knees with more severe damage (KL4), suggesting a dynamic role for the synovium in the disease process [43]. Data from the osteoarthritis initiative concluded that a higher BMI was associated with a higher prevalence and severity of synovial inflammation [44]. Taken together, these findings reinforce our observation that obese patients are at a higher risk of more severe KOA, as synovial *GDF5* expression correlates with disease severity and synovitis is more prevalent in individuals with higher BMI.

This is the first study to reflect the reality of a tertiary public hospital in a developing country. The institute is renowned for its diagnosis and treatment of complex knee diseases, and its care of patients in the Brazilian public system now includes epidemiological research and the identification of genetic biomarkers. However, because tertiary centers typically receive more severe or complex cases, and this is a single-center study with a modest sample size and no replication or ancestry adjustment, the characteristics of the patients treated in this setting may also introduce a selection bias, which should be considered when interpreting our findings. Additionally, the lack of genomic ancestry adjustment may lead to residual population stratification, particularly given the known variation in *GDF5* allele frequencies across different ancestries. However, all individuals came from the same region of Brazil, had similar social backgrounds, and were recruited from the same public hospital. Therefore, no significant racial differences are expected among KOA groups, as they all had equal access to the public health system and the same treatment. Nevertheless, some negative aspects should be pointed out, such as the multifactorial nature of the disease, which can influence the analysis of clinical data, and the fact that most patients wait for long periods before starting adequate treatment for KOA. Moreover, given the cross-sectional design, the associations observed cannot establish temporality or causality. In addition, the modest height difference (~3 cm) between genotypes may be influenced by environmental and socioeconomic factors, and small differences for this SNP have been reported previously [32], highlighting the need for longitudinal or more controlled studies to confirm its effect. Although this is basic research, the results of this study can be applied to future research, given that mixed-race populations are underrepresented in genetic and clinical databases. As well as presenting comprehensive clinical and epidemiological data on a representative sample of patients with KOA, we also present relevant genetic information on these patients. The main positive aspect is the prospective collection of both clinical data and biological samples for analysis, which allows for paired evaluation and minimizes the risk of bias due to recall data. All patients were evaluated by experienced orthopedic surgeons who specialize in knee conditions. These surgeons confirmed the diagnosis and excluded other knee disorders. Our findings help to improve our understanding of the genetic architecture of KOA in mixed-race populations. Integrating this genetic information into clinical assessments may enhance the evaluation of KOA severity and support the development of more personalized clinical management strategies.

## 5. Conclusions

The observed association between the presence of at least one *GDF5* rs143384 A allele and greater severity of KOA and shorter stature among female patients suggests that this variant may contribute to clinical variability and disease progression in knee osteoarthritis.

## Figures and Tables

**Table 1:** Epidemiological and clinical profile of the KOA patients included in the study, presented overall and stratified by severity (n = 224).

**Characteristics**	**All Patients (n = 224)**	**Grades I–III (n = 145)**	**Grades IV–V (n = 79)**	***p*-Value ^a^**	**OR (95% CI) ^b^**
**Age (years) ^d^**	n (%)	n (%)			
<58	46 (20.5)	39 (26.9)	7 (8.9)	<0.001	1 ^c^
58–64	74 (33.0)	50 (34.5)	24 (30.4)		3.14 (1.20–8.27)
65–70	53 (23.7)	36 (24.8)	17 (21.5)		2.86 (1.04–7.90)
>70	51 (22.8)	20 (13.8)	31 (39.2)		9.34 (3.35–26.01)
**Sex**					
Female	170 (75.9)	114 (78.6)	56 (70.9)	0.16	1 ^c^
Male	54 (24.1)	31 (21.4)	23 (29.1)		1.66 (0.82–3.35)
**Stature (meters)**					
<1.54	45 (20.1)	22 (15.2)	23 (29.1)	0.35	1 ^c^
1.54–1.59	69 (30.8)	46 (31.7)	23 (29.1)		0.67 (0.29–1.54)
1.60–1.66	58 (25.9)	41 (28.3)	17 (21.5)		0.51 (0.20–1.31)
>1.66	52 (23.2)	36 (24.8)	16 (20.3)		0.36 (0.12–1.13)
**BMI (kg/m^2^)**					
≤29.9	73 (32.6)	42 (29.0)	31 (39.2)	0.10	1 ^c^
30–35	72 (32.1)	53 (36.6)	19 (24.1)		0.59 (0.28–1.25)
>35	79 (35.3)	50 (34.4)	29 (36.7)		1.37 (0.65–2.89)
**Trauma**					
No	211 (94.2)	133 (91.7)	78 (98.7)	0.16	1 ^c^
Yes	13 (5.8)	12 (8.3)	1 (1.3)		0.22 (0.03–1.82)
	Female KOA patients (n = 170)				
**Characteristics**	**All Patients (n = 170)**	**Grades I–III (n = 114)**	**Grades IV–V (n = 56)**	***p*** **-Value ^a^**	**OR (95% CI) ^e^**
**Age (years) ^d^**	n (%)	n (%)			
<58	36 (21.2)	30 (26.3)	6 (10.7)	0.004	1 ^c^
58–64	56 (32.9)	38 (33.3)	18 (32.1)		2.95 (1.01–8.59)
65–70	43 (25.3)	30 (26.4)	13 (23.3)		2.72 (0.89–8.35)
>70	35 (20.6)	16 (14.0)	19 (33.9)		8.18 (2.56–26.16)
**Stature (meters)**					
<1.54	45 (26.5)	22 (19.3)	23 (41.1)	0.43	1 ^c^
1.54–1.59	63 (37.0)	45 (39.5)	18 (32.1)		0.61 (0.26–1.46)
1.60–1.66	45 (26.5)	36 (31.6)	9 (16.1)		0.44 (0.16–1.24)
>1.66	17 (10.0)	11 (9.6)	6 (10.7)		0.86 (0.25–2.99)
**BMI (kg/m^2^)**					
≤29.9	44 (25.9)	29 (25.4)	15 (26.8)	0.20	1 ^c^
30–35	53 (31.2)	39 (34.2)	14 (25.0)		0.87 (0.35–2.15)
>35	73 (42.9)	46 (40.4)	27 (48.2)		1.73 (0.74–1.13)
**Trauma**					
No	161 (94.7)	105 (92.1)	56 (100.0)	0.99	1 ^c^
Yes	9 (5.3)	9 (7.9)	0 (0.0)		-

BMI: body mass index, CI: confidence interval, OR: Odds ratios, ^a^ *p*-value ≤ 0.05 was obtained through the Chi-square test (Pearson’s *p*-value). ^b^ OR adjusted by age, sex, BMI, and trauma. ^c^ Reference group. ^d^ Continuous variable was categorized by the quartiles. ^e^ OR adjusted by age and BMI.

**Table 2:** Association analysis between *GDF5* rs143384 polymorphism and Ahlbäck classification in female patients with KOA (n = 170).

***GDF5*** **rs143384 G > A**	**Grades I–III (n = 114)**	**Grades IV–V (n = 56)**	***p*-Value ^a^**	**OR (95% CI) ^b^**
	n (%)			
GG	48 (42.1)	13 (23.2)	0.05	1 ^c^
GA	43 (37.7)	30 (53.6)		2.73 (1.21–6.18)
AA	23 (20.2)	13 (23.2)		2.03 (0.76–5.40)
GA + AA	66 (57.9)	43 (76.8)	0.02	2.48 (1.16–5.33)
***GDF5*** **rs143384 G > A**	**Grades I–II** **(n = 84)**	**Grades III–V** **(n = 86)**	***p*** **-Value ^a^**	**OR (95% CI) ^b^**
	n (%)			
GG	37 (44.0)	24 (27.9)	0.09	1 ^c^
GA	31 (36.9)	42 (48.8)		2.25 (1.06–4.79)
AA	16 (19.1)	20 (23.3)		1.98 (0.79–4.96)
GA + AA	47 (56.0)	62 (72.1)	0.03	2.16 (1.08–4.33)
***GDF5*** **rs143384 G > A**	**Grades I–II** **(n = 84)**	**Grades IV–V** **(n = 56)**	***p*** **-Value ^a^**	**OR (95% CI) ^b^**
	n (%)			
GG	37 (44.0)	13 (23.2)	0.09	1 ^c^
GA	31 (36.9)	30 (53.6)		2.66 (1.11–6.37)
AA	16 (19.1)	13 (23.2)		2.08 (0.71–6.06)
GA + AA	47 (56.0)	43 (76.8)	0.03	2.47 (1.09–5.58)

CI: confidence interval, OR: Odds ratios, ^a^ *p*-value ≤ 0.05 was obtained through the Chi-square test (Pearson’s *p*-value). ^b^ OR adjusted by age and BMI. ^c^ Reference group.

## Data Availability

The data presented in this study are available on request from the corresponding author.

## Abbreviations

The following abbreviations are used in this manuscript:

BMI	Body mass index
CI	Confidence intervals
GDF5	Growth differentiation factor 5
GWAS	Genome-wide association studies
HWE	Hardy–Weinberg equilibrium
IQR	Interquartile range
KOA	Knee osteoarthritis
NIH	National Institutes of Health
OR	Odds ratio
PCR	Real-Time Polymerase Chain Reaction
SNP	Single nucleotide polymorphism
